# Awe Narratives: A Mindfulness Practice to Enhance Resilience and Wellbeing

**DOI:** 10.3389/fpsyg.2022.840944

**Published:** 2022-04-14

**Authors:** Jeff Thompson

**Affiliations:** Department of Psychiatry, Columbia University Irving Medical Center, New York, NY, United States

**Keywords:** awe, awe experiences, wellbeing, resilience, mental health, narrative, mindfulness

## Abstract

It is necessary to have available a variety of evidence-based resilience practices as we experience life’s stressors including the ongoing COVID-19 pandemic. Evoking, experiencing, and reflecting on awe moments by developing and sharing an “awe narrative” are a type of mindfulness technique that can have the potential to help someone flourish, enhance their resilience, and have a positive impact on their overall wellbeing. This paper explores how constructing an awe narrative can assist the individual while also possibly having a positive impact on others.

## Introduction

Part of the human condition involves experiencing stress and other emotions such as fear and anxiety. When not managed properly, these can have a detrimental impact on mental health. Therefore, it is necessary to develop evidence-based and practical techniques in order to maintain and enhance one’s mental health and resilience. Reflecting on the phenomenon of awe and one’s personal experience of it by developing and sharing a narrative is one such mindfulness practice that can provide an important counterbalance that can allow one to endure and thrive during the ongoing COVID-19 global pandemic as well other life stressors and negative events. Developing practices in resilience and wellbeing can create that necessary counterbalance, as reports have stated that COVID-19 can have potential negative effects on mental health (Czeisler et al., 2020; Dawel et al., 2020; Brülhart et al., 2021).

### Mindfulness Literature

Mindfulness is a type of awareness that occurs from purposefully paying attention in the present moment while doing so non-judgmentally (APA, 2012; Mindful, 2017). The beneficial outcomes of mindfulness include it can increase hope and optimism, quality of life, connectedness, and overall positivity (Vago and Silbersweig, 2012). Numerous studies have demonstrated how a variety of mindfulness practices can enhance an individual’s resilience (Pidgeon and Keye, 2014; Southwick and Charney, 2018; Kachadourian et al., 2021; Linder and Mancini, 2021). Mindfulness practices can include deep breathing, meditation, walking, body scanning, and both listening and talking (Ackerman, 2021; Moore, 2022).

Experiencing awe has been associated with being both a resilience practice as well as being referred to as a type of mindfulness practice (Lutz et al., 2015; Keltner, 2017; Clark, 2020; Sturm et al., 2020; Tabibnia, 2020; Büssing, 2021). According to researcher Michelle “Lani” Shiota, experiencing awe can be a temporary form of mindfulness that can be easier to experience compared to other mindfulness practices that require training because awe can be felt automatically (D’Ardenne, 2019). Specifically, and for the scope of this paper, awe narratives are considered a type of mindfulness practice as it can captivate a person in the moment drawing their full attention—through reflecting on their own narrative or being exposed to someone else’s awe narrative (Rudd et al., 2012; Piff et al., 2015; Keltner, 2016; Clark, 2020). This type of awareness is a significant element to both mindfulness and experiencing awe (Rudd et al., 2012; Kabat-Zinn, 2015; Moore, 2022).

### Resilience Literature

Numerous researchers have defined the concept of “resilience.” The American Psychological Association, 2020 for example, explains it as “the process of adapting well in the face of adversity, trauma, tragedy, threats, or significant sources of stress.” The APA further explains that resilience is more than merely “bouncing back” and involves personal growth beyond the adverse experience. To expand on this definition, the author defines resilience as taking ongoing, proactive measures to sustain and enhance one’s mental and physical health. Resilience involves using those practices to bounce back from difficult, adverse situations. Importantly, resilience is also reaching out for help when it is needed.

There are numerous evidence-based resilience practices people can utilize in their everyday lives. Many articles and books have been written about enhancing and sustaining resilience, and one might begin by reviewing the article by Tabibnia and Radecki (2018) as well as the book by Southwick and Charney (2018). Briefly, some of the practices involved include the following: cognitive reappraisal, controlled breathing and meditation, emotional regulation, gratitude, meaning and purpose in life, mindfulness, optimism, physical exercise, self-compassion, social connectedness, spirituality and faith, and sufficient sleep. Furthermore, eliciting awe and reflecting on moments of awe is an additional practice of resilience (Tabibnia, 2020).

### Awe Literature

Awe has been described as an emotion that is evoked during an experience involving a sense of vastness, while also going beyond one’s typical comprehension of expectations, thus creating a “need for accommodation”—a need to further understand it (Keltner and Haidt, 2003).

Awe can be experienced in a variety of ways, including nature, space, art, music, religious and spiritual moments, another’s or your own accomplishments, and social interactions (Shiota et al., 2007; Pilgrim et al., 2017; Allen, 2018; Anderson et al., 2018; Graziosi and Yaden, 2019). Awe has been elicited in various environments, including in person in settings such as nature (Anderson et al., 2018), laboratory settings (Gallagher et al., 2014), and through virtual reality (Chirico et al., 2017).

Research has shown that experiencing awe can have numerous positive benefits, such as increasing prosocial behaviors (Piff et al., 2015) including sharing (Piff et al., 2015) and kindness (Bai et al., 2017), while it can also enhance learning (Krogh-Jespersen et al., 2020), interconnectedness (Shiota et al., 2007; Yang et al., 2018), creativity, humility, gratitude and optimism (Nelson-Coffey et al., 2019), openness, and critical thinking (Stellar et al., 2018). Research has also shown that experiencing awe can be beneficial for both our physical and mental health (Stellar et al., 2015) and our overall wellbeing (Rudd et al., 2012).

Experiencing awe can also make a person feel small in a good way (Shiota et al., 2017; Allen, 2018; van Elk et al., 2019). A positive impact of feeling small is that this can reduce a person’s focus on themselves as well as reduce their personal concerns (Reinerman-Jones et al., 2013; Piff et al., 2015; Elk et al., 2016). Although awe and mindfulness have different mental states, with respect to smallness, experiencing both awe and mindfulness can produce a reduced sense of the self while also feeling connected to others (Yaden et al., 2017).

### Eliciting Awe Through Narratives

For the purposes of this paper, elicitors and the corresponding impact of awe, as well as other emotions associated with awe, will be detailed, including nature, personal accomplishment, smallness, and social connectedness. Specifically, a first-person narrative is provided to illustrate a type reflective practice that can further elicit awe and enhance one’s mental health and resilience. Studies have shown that recalling and sharing personal experiences are a known method for studying (Shiota et al., 2017; Allen, 2018) and cultivating awe (Bai et al., 2017; Danvers and Shiota, 2017; Stellar et al., 2018; Chen and Mongrain, 2020).

Research has shown that this type of reflective practice can be effective in enhancing the wellbeing of the individual (East et al., 2010; Piff et al., 2015; Shiota et al., 2017). Moreover, eliciting awe can occur not only through sharing one’s own experiences, but also through learning about the experiences of others (Rudd et al., 2012; Piff et al., 2015; Cuzzolino, 2021), including when hearing about the success stories of others (Walker and Gilovich, 2021).

### Awe Narrative

This is a story about awe. I live in a major city, which means there are lots of large buildings and many people coming and going quickly either on foot or by bus, train, and car. Here, I was at a conference many hours away in a suburb of a much smaller city. On the last day of the conference, I noticed outside the window, as I had each day previously, a big mountain—“big” at least according to my city standards! I went on Google maps, looked up the name of the mountain, and searched if one could hike or climb it (same thing, right?). I smiled with excitement as I discovered one could, and before I knew it, I had changed into hiking clothes (which were really my gym clothes) and was in a taxi on my way there.

I had two options: take the easier route or the more challenging path to the top. I chose the latter. The solo trek to the top took just over 2 h. Although I embarked on my trek alone, I exchanged frequent “hellos” with other hikers I passed on the way up and had similar pleasant exchanges with those on their way down.

To put it nicely, I was (and still am) an amateur hiker. Although not overbearing, the journey certainly had its difficult moments. At a certain point near the top, I stopped to look out, far out, at the vast view I now yearned to take in. I took a deep breath. I felt something pass through my entire body—and not just on my skin: it was much deeper. That feeling is impossible to further describe, but the sensation was one of calm, which at the same time included a small dose of fear, as I was a mere four feet from a ledge that, in my mind, was thousands of feet above ground.

I saw trees. I saw empty swaths of land. I saw hundreds of houses neatly aligned on grid-like streets. I noticed cars driving to unknown destinations and at that moment I thought, “There they are and here am I.” Then, I realized that the cars looked like specks moving on the ground and that to the people in them I probably was not even a speck. Everything, including myself, seemed so small; yet as I also looked around me there certainly was vastness—everywhere.

I looked around again at this real-life panoramic view and although I am still not sure if I said this out loud or only in my head, certainly in one form or another I quietly uttered “Woooow.”

Having played sports most of my early and adult life, the feeling was similar, in retrospect, to winning a big game, yet also very different. I felt as if I would definitely won, but I had not “beaten” or “defeated” anyone. Instead, in this glorious victory, I felt I was somehow more connected. More connected to whom? To the people at the top and to those behind me—in fact to people and things everywhere. Everywhere and everything. Instead of a trophy, my prize was feeling that “Woooow” sensation, along with an “Ahhhh.”

I felt like high-fiving someone for no apparent reason. I knew how long exactly it had taken me, as my watch had timed my hike, yet while I had reached the summit, at this moment all sense of time escaped me. Not that time stood still, but more accurately, if it makes sense, I was beyond time. I was in that moment completely without past or future. I was completely present.

The air was energizing and felt and smelled refreshing as I breathed it in. I took another breath; in reality, I most likely took a few, and these were a bit longer than the normal inhale and exhale. Again, in retrospect, without realizing it at the moment, I literally was trying to take it all in.

My view was all-encompassing: 360 degrees of life, colors, objects, and nature everywhere. The sky was various shades of blue. The scattered white clouds cast impressive shadows on the neighborhoods below. My eyes fixed on some trees that, from my perspective, were smaller than my fingernail. Yet, were I standing down there next to them, they certainly would be at least five times my size. The mountain in the distance had a distinct, sharp, and dark orange-brown glow at its base that transitioned to a subdued tan the higher one looked toward it peak. Where the land eventually met the sky at the horizon had to be many hundreds of miles away.

Here, then, was little me, seeing it all and realizing how much the “all” really includes. I felt small. Small but not inferior, as “little me” was also connected to everything I beheld.

As I walked around at the top of this mountain, I saw plenty of other fellow trekkers in groups or alone taking it all in, just as I was. I looked out and again beheld the vastness and experienced a new view while seeing something I recognized-I saw my hotel. The previous day, the view was the exact opposite. I was at that hotel standing outside my room and leaning on the outdoor walkway railing looking up at this same exact mountain.

However, now here I am on top of the mountain, looking out at the hotel I was staying in, and although I could not see if someone was standing outside and leaning on the railing as I had the day before, I thought to myself, “If someone is standing there looking this way and contemplating venturing up this mountain, I hope they make the same decision I did: it’s worth it.”

Although I was a safe distance from the edge, others were much closer to it. I did not like this, not one bit. I then looked down. Bad move. You know that sensation people have when taking a plunge on a rollercoaster? Well, that is exactly what I experienced on that mountain: the view below literally took my breath away. I calmly (well, as calmly as possible) turned around to regain it.

Then, I noticed something new. There, at the top of the mountain, was a small tree or bush. Perhaps a tree-bush, if there is such a thing (I am not known for my comprehensive knowledge of botanical terminology). Just as I had been taking in a sprawling, thousand-mile, 360-degree view, I now became fixated on this roughly four-by-four-foot shrub.

I was here, at the peak of this immense mountain, and so too was this little tree. A little tree that made it up here years before me and managed to live and thrive. It adapted, too, in order to do so. Many of its roots were exposed and grew around the surrounding rocks. I wondered: How does that even happen? How did it know to do that?

As much as the ever-expanding views were breathtaking, this view was equally impressive.

As I continued to absorb everything, I felt as if all of my senses were being utilized. Not in an overwhelming way, mind you, but as a fully immersive experience. I felt on my forehead the warm, gentle breeze that was also tinged with coolness, to the point that I now had slight goose bumps on my arms. I noticed everything I looked at and realized I was closer to the clouds than to everything going on at ground level. I saw the details of the clouds and, at my feet, the various colors of the dirt mixed with clay.

I took a sip of water. Although I was not gasping for it, I knew it was needed. As I drank, I felt the water make its way from my mouth through my throat and eventually into my stomach. Although I have had many a glass of water in the past, this was unlike any other water-drinking experience.

All was silent as my gaze went from looking at things nearby, to scanning the horizon, to observing everything in between. Yet, at the same time I also heard people talking in tones of joy and excitement, as clearly, they were having their own independent experience, which I, a stranger, was also sharing. We were strangers connecting together.

I now took a deep, purposeful breath and felt my chest expand. The only thing missing was to place my hands on my hips and pretend I was Superman. Indeed, this moment did feel “super.” I smiled as I thought to myself, “I did it. I’ve earned this. I’ve just climbed this mountain. I am seeing this because I chose to climb it. And now here I am with others enjoying their own experiences and sensing their own accomplishment in their own way—and we are all together.”

I realized then that this must be awe.

I took another deep breath, exhaled, and looked around. I smiled again, this time in gratitude. I then began my return journey down the mountain.

## Discussion

Awe is an emotion that can be elicited in a variety of ways, and as a mindfulness practice, it can have numerous positive benefits on an individual’s wellbeing while also potentially enhancing their resilience (Rudd et al., 2012; Piff et al., 2015; Shiota et al., 2017; Yang et al., 2018; Tabibnia, 2020). Table 1 exhibits the relationship between what previous research has shown to be the attributes and benefits of experiencing awe with specific examples of how each were demonstrated in the narrative. The third column expands the relationship in the table by further demonstrating how each are connected to resilience.

**Table 1:** Awe: Linking the research with the narrative and resilience.

Potential attributes and benefits of awe	Narrative example	Connection to resilience
Accomplishment	*I smiled as I thought to myself, “I did it. I’ve earned this. I’ve just climbed this mountain. I am seeing this because I chose to climb it*.	Promotes self-efficacy and contributes to overall wellbeing and meaning in life (Bandura et al., 1997).
Connectedness (with others)	*I felt I was somehow more connected…To the people at the top and to those behind me—in fact to people and things everywhere*.	Positive influence on mental and physical health, contributes to positive behaviors, and manage stress (Southwick and Charney, 2018).
Connectedness (with nature)	*The mountain in the distance…Where the land eventually met the sky at the horizon had to be many hundreds of miles away… connected to everything I beheld*.	Linked with personal resilience (Inguili and Lindbloom, 2013).
Cognitive Reappraisal	*…small dose of fear, as I was a mere four feet from a ledge that, in my mind, was thousands of feet above ground… Although I was a safe distance from the edge, others were much closer to it. I did not like this, not one bit. I then looked down. Bad move…* *I smiled… I am with others enjoying their own experiences and sensing their own accomplishment in their own way—and we are all together*.	Managing stress and emotions, overall wellbeing, and life satisfaction (Xu et al., 2020).
Curiosity	*I live in a major city… I noticed outside the window, as I had each day previously, a big mountain… I went on Google maps, looked up the name of the mountain, and searched if one could hike or climb it*.	Improves happiness, meaning in life, hope, and strengthens relationships (Campbell, 2015; Wolff, 2019).
Gratitude	*I took another deep breath, exhaled, and looked around. I smiled again, this time in gratitude*.	Stress management, self-efficacy, physical health, and social connections (Emmons 2010; Millstein et al., 2016).
Need for accommodation	*I was here, at the peak of this immense mountain, and so too was this little tree. A little tree that made it up here years before me and managed to live and thrive. It adapted, too, in order to do so. Many of its roots were exposed and grew around the surrounding rocks. I wondered: How does that even happen? How did it know to do that?*	Contributes to cognitive flexibility, handling uncertainty and ambiguity, and contributes to cognitive flexibility (Iacoviello and Charney, 2014; Southwick and Charney, 2018).
Smallness	*Here, then, was little me, seeing it all and realizing how much the “all” really includes. I felt small. Small but not inferior, as “little me” was also connected to everything I beheld*.	Cognitive reappraisal; reduces personal concerns; humility is associated with personal resilience (Piff et al., 2015; Devenish- Meares, 2016; Martin and Ochsner, 2016).
Social interactions and prosocial behaviors	*I exchanged frequent “hellos” with other hikers I passed on the way up and had similar pleasant exchanges with those on their way down… I felt like high-fiving someone… We were strangers connecting together*.	Counters loneliness, promotes happiness, and contributes to overall wellbeing and resilience (Baumeister et al., 2013; Titova and Sheldon, 2021).
Time “slowing down”	*…at this moment all sense of time escaped me. Not that time stood still, but more accurately, if it makes sense, I was beyond time. I was in that moment completely without past or future. I was completely present*.	Mindfulness; enhances decision making, and wellbeing (Rudd et al., 2012).
Tolerate ambiguity and fear	*That feeling is impossible to further describe, but the sensation was one of calm, which at the same time included a small dose of fear, as I was a mere four feet from a ledge that, in my mind, was thousands of feet above ground*.	Reframe the situation to see it differently and more positively (see cognitive reappraisal); being able to handle the uncertainty (Southwick and Charney, 2018).
Vastness	*I saw trees. I saw empty swaths of land. I saw hundreds of houses neatly aligned on grid-like streets… I also looked around me there certainly was vastness—everywhere*.	Increases connectedness and smallness (see above).

Experiencing awe is closely linked with resilience and wellbeing, and with the narrative shared attempts to demonstrate how various emotions can overlap during a single event, which is also the case for various resilience concepts and practices. These emotions, aside from awe, include happiness, joy, astonishment, wonder, accomplishment, surprise, and contentment.

The various resilience concepts in the above mindfulness experience, as detailed in Table 1, include self-transcendence, cognitive reappraisal and self-efficacy, or believing in one’s abilities.

Self-transcendent experiences (STEs) are described as feeling a sense of unity and a connection “with other individuals, humankind, and even the entirety of existence” (Yaden et al., 2017, p. 5). Experiencing awe and STEs have been closely linked together (Yaden et al., 2017; Chirico and Yaden, 2018; Li et al., 2019; Jiang and Sedikides, 2021) and in this narrative, it was experienced at numerous moments (for examples, see in Table 1, *connectedness with others and with nature*).

STEs is central to one’s health (Wong, 2016; Liu et al., 2021) and it can result in prosocial behavior, which is especially important during the COVID-19 pandemic (Waters et al., 2021). Aside of it helping develop strong social connections, helping others have shown increases in personal happiness and having meaning in life (Baumeister et al., 2013; Titova and Sheldon, 2021). Expressing gratitude, another prosocial behavior, also has been shown to increase resilience (Emmons 2010). Prosocial behaviors are also particularly important during COVID-19 as they can counter feelings of loneliness, which has been labeled an epidemic (Health Resources and Services Administration, 2019), can be detrimental to one’s health (Novotney, 2019).

Additionally, Paersch et al. (2021) has demonstrated how one’s resilience can be enhanced through sharing self-efficacy narratives, specifically with respect to being efficient at cognitive reappraisal, or being able to reassess a negative or stressful situation by also finding something positive about it. Cognitive reappraisal is an important practice in resilience (Ochsner et al., 2009 Troy and Maus, 2011 Martin and Ochsner, 2016; Southwick and Charney, 2018).

As individuals try to maintain their mental health during this global pandemic and emerge into a “new normal,” it is imperative that new, innovative, and practical resilience practices evolve. Research has shown that both reflecting on personal awe moments and reading other people’s accounts of awe experiences can elicit awe and support one’s wellbeing. Further, the awe narrative shared in this paper has shown that an awe experience can also be linked to numerous other resilience practices and skills and thus, it can be supportive of an individual’s overall wellbeing.

### Future Directions and Limitations

One should note that readers might not necessarily experience or agree with some, all, or any of the above-mentioned emotions or concepts explained as forming part of the awe story. After all, when awe is viewed and studied as a phenomenon, it is the individual experience of awe that is being examined, not how others (readers) interpret that person’s experience, regardless if that interpretation is the same as theirs or not. The essence of phenomenology is to understand the reality of a phenomenon as it is lived by a person (van Manen, 1990; Neubauer et al., 2019).

Therefore, future studies should be conducted on awe narratives further examining the positive impact both personal and the awe experiences of others can have on individuals. For example, by exposing participates to this awe narrative, a study can gauge if the same experience of awe and related resilience attributes are felt with the reader as it was with the person who experienced it first-hand. Further, studies can examine if there is a difference between participates reading the narrative compared to listening to it narrated *via* an audio file.

Reflecting on personal, previous awe moments and those of others are also just one of numerous examples of how awe can be elicited, even when restrictions are in place such as it frequently has occurred during COVID-19. Technology can be continued to be embraced to utilize awe experiences and other mindfulness practices to support an individual’s resilience. For example, a mobile and online accessible cohort-based program that was designed to support personal resilience which includes awe and other mindfulness practices has had promising, preliminary results (Thompson, 2020; Thompson and Drew, 2020). This includes feelings of connectedness, which is especially critical to combat growing concerns of the rise in loneliness as a result of COVID-19 (Weissbourd et al., 2021; Chokshi, 2022).

Additional options to support this, especially during COVID-19, include through virtual and augmented reality, watching videos, listening to music, and viewing images (for additional examples, see Shiota and Greater Good Science Center, 2016; Magnan, 2020; Fessell and Reivich, 2021; Thompson, 2022).

## Conclusion

Given the stressors individuals experience in a lifetime, including those generated by the current COVID-19 pandemic and other factors, a mindfulness practice of reflecting on moments of awe and developing narratives from those moments can potentially contribute to maintaining and enhancing the wellbeing and resilience of both the person reflecting on his or her experience as well as the people that experience is shared with.

## Data Availability Statement

The original contributions presented in the study are included in the article/supplementary material, further inquiries can be directed to the corresponding author.

## Author Contributions

The author confirms being the sole contributor of this work and has approved it for publication.

## Conflict of Interest

The author declares that the research was conducted in the absence of any commercial or financial relationships that could be construed as a potential conflict of interest.

## Publisher’s Note

All claims expressed in this article are solely those of the authors and do not necessarily represent those of their affiliated organizations, or those of the publisher, the editors and the reviewers. Any product that may be evaluated in this article, or claim that may be made by its manufacturer, is not guaranteed or endorsed by the publisher.
